# A framework for slow physiological motion compensation during HIFU interventions in the liver: proof of concept

**DOI:** 10.1186/2050-5736-3-S1-P54

**Published:** 2015-06-30

**Authors:** Cornel Zachiu, Baudouin Denis de Senneville, Sjoerd Crijns, Bas Raaymakers, Chrit Moonen, Mario Ries

**Affiliations:** 1 University Medical Center Utrecht, Utrecht, Netherlands

## Background/introduction

While respiratory motion compensation for HIFU interventions for liver cancer therapy has been extensively studied, the influence of slow physiological motion, such as peristalsis, has so far been largely neglected. During the lengthy intervention, the magnitude of the latter can exceed acceptable therapeutic margins and lead to a substantial mismatch between planned ablation volume, thermal dose estimates and the measured non-perfused volume (NPV). Given the episodic nature of a HIFU intervention, this study proposes the integration of a 3D motion compensation procedure based on MR-images for slow physiological motion and validates the approach on *in vivo* ablations on a porcine liver.

## Methods

### Overall strategy

A volumetric HIFU ablation was completed over a time span of 2h using a Phillips Sonalleve system with a respiratory gating strategy for both energy delivery and all MR-imaging. A 3D image was acquired before the first sonication, as well as after each sonication (Δt=5min) to track slow physiological motion. The estimated motion fields were used to: 1) Estimate on the planning image the position of the true ablated anatomy; 2) Register the temperature maps into the initial reference position in order to compute a correct thermal dose estimate, and 3) Register the NPV to the initial position.

### Assessment of 3D liver displacements

Liver displacements were estimated using an optical flow algorithm applied on 3D MR images.

### MR imaging protocol

3D T1-weighted images were acquired on a 1.5T Philips Achieva MR scanner (Philips Healthcare, Best, The Netherlands) using the following protocol: TE=2ms, TR= 4.3ms, matrix size=192x192x75, FA=10°, voxel size=2x2x2mm3.

## Results and conclusions

**Figure 1:**
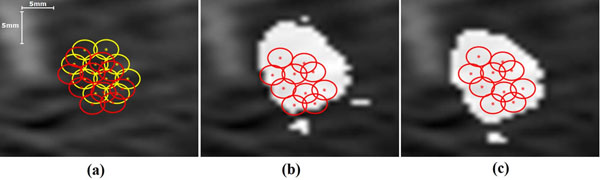
Planned (yellow) and motion corrected (red) sonication cluster overlaid on the corresponding anatomy in the planning image. The hyper-intense regions represent the (b) non-corrected (c) corrected location of the anatomy that receives a lethal thermal dose

Motion tracking revealed an initial shift of up to 4mm during the first 10min, which is most likely caused by the muscle relaxant effect of anesthesia and a subsequent continuous shift due to bowel gas development of ~2mm until the end of the intervention. This leads to a continuously increasing mismatch of the initial shot planning, the thermal dose measurements and the true underlying anatomy as shown in Fig. 1a, 1b. The estimated displacements allowed correcting the planned sonication cell cluster positions to the true target position (Fig. 1a), as well as the thermal dose estimates (Fig. 1c) and the NPV-measurement (data not shown). A spatial coherence of all three is particularly important to assure a confluent ablation volume and to prevent remaining islets of viable malignant tissue. The *in vivo* experiment also demonstrates that the proposed framework is compatible with the work-flow of a HIFU intervention under clinical conditions.

